# Retina and microvascular alterations in migraine: a systemic review and meta-analysis

**DOI:** 10.3389/fneur.2023.1241778

**Published:** 2023-09-28

**Authors:** Ziqiang Liu, Chuanhong Jie, Jianwei Wang, Xiaoyu Hou, Weiqiong Zhang, Jingying Wang, Yu Deng, Yuanyuan Li

**Affiliations:** Eye Hospital China Academy of Chinese Medical Sciences, Beijing, China

**Keywords:** migraine, microvascular alterations, retinal nerve fiber layer, meta-analysis, OCTA, OCT

## Abstract

**Objective:**

This study aimed to evaluate the retina and microvascular alterations with optical coherence tomography (OCT) or optical coherence tomography angiography (OCTA) in patients with migraine with aura (MA) and migraine without aura (MO).

**Methods:**

PubMed, Embase, and Cochrane Library databases were searched to find relevant literature on patients with MA or MO using OCT/OCTA devices. The eligible data were analyzed by Stata Software (version 15.0).

**Results:**

There were 16 studies identified, involving 379 eyes with MA, 583 eyes with MO, and 658 eyes of healthy controls. The thickness of the peripapillary retinal nerve fiber layer (pRNFL) of patients with MA decreased significantly in most regions. The foveal avascular zone (FAZ) area and perimeter in MA patients significantly enlarged, while the perfusion density (PD) in the macular deep capillary plexus (mDCP) significantly decreased in the whole image and its subregions except for the fovea, with the PD in radial peripapillary capillary (RPC) decreasing inside the disk. Patients with MO demonstrated a significantly decreased thickness of pRNFL in most regions, and the FAZ parameters were significantly enlarged. No statistical significance was observed in the retina and microvascular features of patients with MA and MO.

**Conclusion:**

The eyes affected by MA and MO demonstrated significantly reduced thickness of pRNFL and enlarged FAZ. Patients with MA showed retinal microvascular impairments, including a decreased PD in mDCP. The OCT and OCTA could detect membrane morphology and circulation status in migraine and might provide the basis for the diagnosis and follow-up of patients with migraine.

**Systematic review registration:**

https://www.crd.york.ac.uk/prospero/, CRD42023397653.

## 1. Introduction

Migraine is a common neurovascular disorder with a prevalence of 15–18% in the population (1). It is clinically characterized by recurrent, mostly unilateral, moderate-to-severe throbbing pain (2). Episodic migraine is clinically classified as migraine with aura (MA) and migraine without aura (MO). Visual aura is reported as the most common aura symptom, often manifesting as flashes of light, dark spots, and decreased visual acuity (3). Previous data were inadequate for the diagnosis and treatment of migraine. Therefore, it is important to determine reliable biomarkers that could be employed for the clinical diagnosis, condition assessment, and prognostic follow-up of patients with migraine.

Although the pathogenesis of migraine is not fully understood, an increasing amount of evidence supports the involvement of the neurovascular system in the development of the disease (4). In 1979, Moskowitz et al. proposed the “trigeminovascular theory,” which suggested that the trigeminal vascular pain pathway is a common pathway that leads to the development of migraine (5). The trigeminovascular system (TGVS) consists of the trigeminal nucleus, the trigeminal ganglion, the trigeminal nerve, and its innervated meningeal and ocular vascular networks. The activation of the TGVS increases the release of neurotransmitters and vasoactive intestinal peptides, which activates injurious receptors that transmit pain signals to the center via the trigeminal nociceptive afferent fibers, resulting in pain (6). It has also been found that during visual aura or migraine attacks, perfusion deficit can be caused by reduced blood flow due to transient vasospasm, which occurs not only in the cranial but also in the ocular vasculature. Note that recurrent migraine attacks can cause permanent damage to the brain and retina (7, 8). Migraine has been reported to be closely associated with various neurovascular eye diseases such as retinal artery occlusion, ischemic optic neuropathy, and glaucoma (9–12). Therefore, it needs to be clarified whether migraine causes ischemia and structural changes in the retina and optic disk, which makes patients with migraine more susceptible to these ocular diseases.

OCT and OCTA can now be used to qualitatively and quantitatively detect the status of the retinal nerve fiber layer (RNFL), retina, and optic nerve papillae circulation. Therefore, they are currently used as clinical biomarkers for various neurological diseases (13, 14). While previous studies on migraine hemodynamics focused on the brain using fMRI (15, 16), several recent clinical studies have reported alterations in the retina and retinal perfusion in patients with migraine, which may provide a way to understand the ocular physiopathology observed with migraine (17, 18).

Although many studies have evaluated and reported retina and microvascular alterations in patients with migraine, different and even contradictory results have been obtained. The present meta-analysis combines the OCT and OCTA results about the retinal structure and perfusion in patients with episodic migraine from different studies to compare the differences between patients with episodic migraine and healthy controls and the differences between the different subtypes of MA and MO.

## 2. Materials and methods

The study followed the Preferred Reporting Items for Systematic Reviews and Meta-Analyses (PRISMA) guidelines (19). The PRISMA checklist is detailed in Supplementary File S1.

### 2.1. Search strategy

PubMed, Embase, and Cochrane Library databases were searched for research published from February 2018 to February 2023. The following subject terms and keywords were used to identify relevant studies: “migraine” AND “optical coherence tomography” OR “optical coherence tomography angiography” OR “OCT” OR “OCTA.” Detailed search strategies are provided in Supplementary File S2.

### 2.2. Inclusion and exclusion criteria

The following inclusion criteria were employed: (1) Patients diagnosed with migraine according to the criteria of the International Classification of Headache Disorders, third version (ICHD-3) (2). The detailed diagnostic criteria for migraine are provided in Supplementary File S3. (3) The OCT or OCTA was used to observe the retina and microvascular alterations.

The following exclusion criteria were applied: (1) absence of any comparative studies; (2) insufficient data for meta-analysis; (3) unmatched study object; and (4) conference abstract.

### 2.3. Data collection and risk of bias assessments

Literature screening was carried out independently by two researchers and cross-checked. In case of any disagreement, it was discussed by the two researchers or settled through third-party arbitration. The data include details on basic trial information, population, location, sex, age, mean migraine duration, attack numbers per month, MIDAS score, OCT/OCTA device, scan sizes, and outcome variables.

This study used the Newcastle–Ottawa Scale (NOS). Two authors evaluated the risk of bias in the included studies from three submissions: selection, comparability, and outcome. All studies were assigned a scale of 0–9.

### 2.4. Data analysis

Statistical analysis was performed using Stata Software version 15.0. We estimated the weighted mean differences (WMDs) and 95% confidence intervals (CIs). The heterogeneity was estimated with *I*^2^ statistics. If the heterogeneity was substantial (*I*^2^ > 50%), a random effect model was employed. Sensitivity or subgroup analysis was used to identify and eliminate the source of the heterogeneity. Descriptive analysis was used if there was still a substantial remaining heterogeneity. Egger’s linear regression test was used to statistically evaluate the potential publication bias.

## 3. Results

### 3.1. Search results

**Figure 1:**
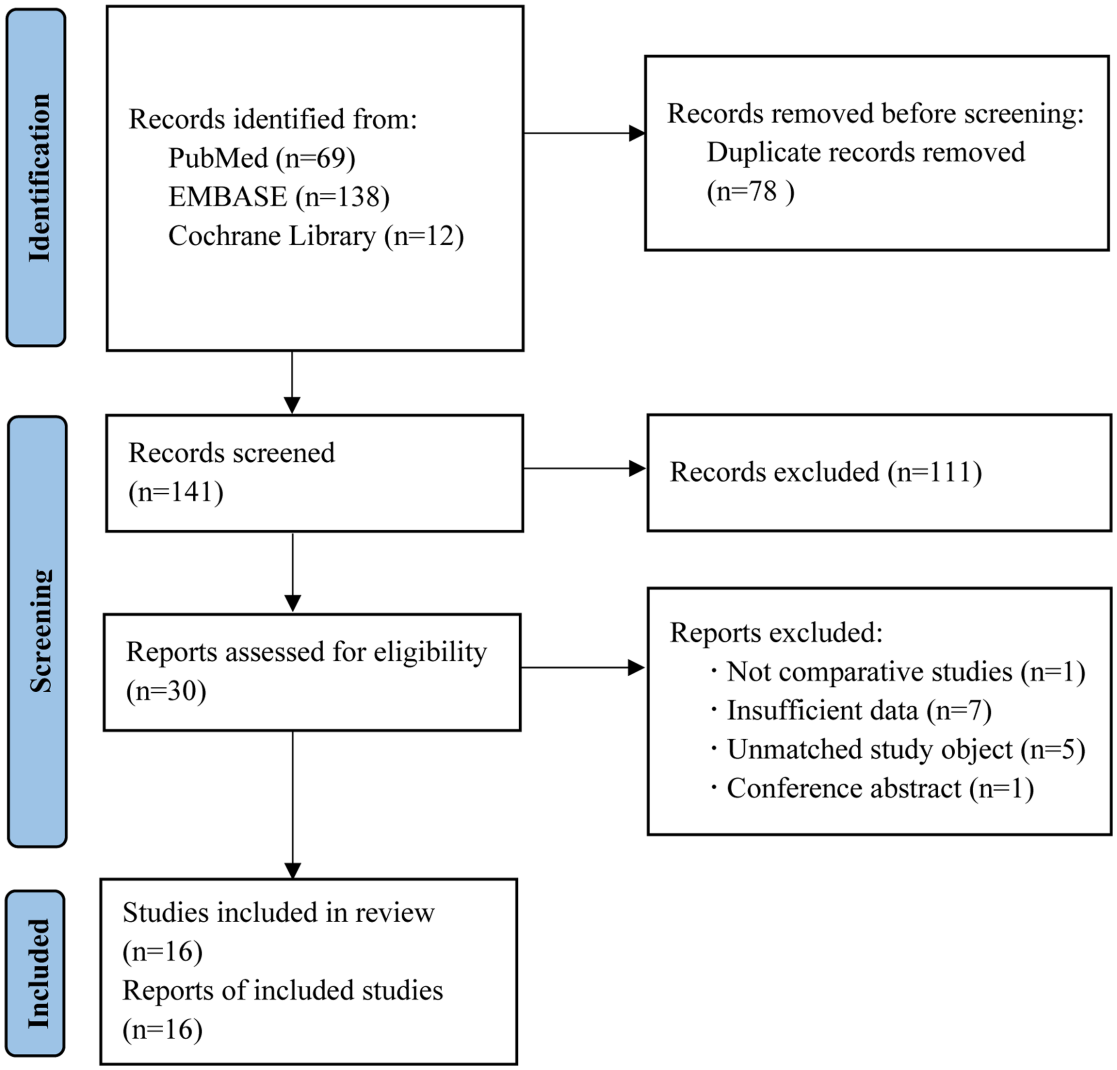
Flow diagram of the literature search process.

We retrieved 219 records from PubMed, Embase, and Cochrane library databases. Among these, 78 duplicates were removed. Among the remaining 141 records, 111 were deleted after reading their titles and abstracts and 14 were removed after closely reading their full text. Finally, 16 studies were selected. The flowchart of the search is shown in Figure 1.

### 3.2. Characteristics and risk of bias assessment of included studies

The final 16 studies had 379 eyes with MA, 583 eyes with MO, and 658 eyes with healthy controls. No significant age- or gender-based difference was found between the groups. All of them reported the type of OCT/OCTA devices used (Zeiss, Optovue, Heidelberg, and Nidek). The detailed characteristics of the studies and the location, disease duration, and outcome variables are listed in Table 1.

**Table 1:** Characteristics of included studies.

Authors (years)	No. of eyes			Location	Mean duration (years)		OCT/OCTA device	Scan Sizes (mm)	Outcome variables	NOS quality score
	MA	MO	HC		MA	MO				
He et al. (18)	32	31	32	China	12.6 ± 9.2	12.6 ± 8.5	Zeiss	M6,O6	FAZ + MPD + RPD	8
Kurtul et al. (17)	-	46	46	Turkey	-	1.32 ± 1.2	Optovue	M6,O4.5	FAZ + MPD + RPD + pRNFL	8
Rego-Lorca et al. (20)	9	8	20	Spain	-	-	Heidelberg	-	pRNFL	8
Temel et al. (21)	-	28	28	Turkey	-	-	Heidelberg	-	RNFL	8
Hamurcu et al. (22)	38	-	38	Turkey	9.47 ± 7.57	-	Optovue	M3,O4.5	FAZ + RPD + pRNFL	7
Dereli et al. (23)	-	108	94	Turkey	-	2.2 ± 1.7	Optovue	M3,O4.5	FAZ + MPD + RPD + pRNFL	8
Karahan et al. (24)	60	-	56	Turkey	-	-	Optovue	M3,O4.5	FAZ + MPD + RPD	7
Hamamci et al. (25)	30	30	30	Turkey	8.92 ± 5.31	6.98 ± 4.81	Optovue	M6,O4.5	FAZ + MPD + RPD + pRNFL	8
Kanar et al. (26)	37	40	50	Turkey	8.05 ± 4.7	9.4 ± 3.9	Zeiss	-	pRNFL	7
Altunisik et al. (27)	28	82	45	Turkey	-	-	Heidelberg	-	RNFL	8
Bingöl et al. (28)	17	16	28	Turkey	-	-	Optovue	M6,O4.5	FAZ + MPD + RPD + pRNFL	8
Güler et al. (29)	-	26	24	Turkey	-	-	Optovue	M6,O6	FAZ + MPD + RPD + pRNFL	8
Taşlı et al. (30)	-	37	43	Turkey	-	7.05 ± 3.46	Nidek	M3,O2.4*4	FAZ + MPD + RPD + pRNFL	7
Ulusoy et al. (31)	28	26	34	Turkey	15.6 ± 4.7	13.7 ± 5.1	Optovue	M6,O4.5	FAZ + MPD + RPD + pRNFL	7
Abdellatif et al. (32)	45	45	40	Egypt	-	-	Nidek	-	pRNFL	8
Ao et al. (33)	55	60	50	China	10.75 ± 7.69	10.39 ± 8.81	Heidelberg	-	pRNFL	7

MA, migraine with aura; MO, migraine without aura; HC, healthy control; OCT, optical coherence tomography; OCTA, optical coherence tomography angiography; M, macular scan; O, optic nerve head scan; FAZ, foveal avascular zone; MPD, macular perfusion density; RPD, radial peripapillary capillary perfusion density; pRNFL, peripapillary retinal nerve fiber layer; NOS, Newcastle–Ottawa scale.

The results of the quality evaluation are shown in Table 1. The results showed that 10 studies scored 8 and 6 studies scored 7, indicating the high quality of the studies. These details are also listed in Supplementary File S4.

### 3.3. Meta-analysis

#### 3.3.1. Meta-analysis for MA versus healthy controls

Table 2 shows the main finding of the meta-analysis on the FAZ, the perfusion density (PD) of the macula superficial capillary plexus (mSCP) and macula deep capillary plexus (mSDP), the PD of the radial peripapillary capillary (RPC), and the thickness of the peripapillary RNFL (pRNFL). When compared to that of the healthy controls, a significant increase was observed in the FAZ area and perimeter in patients with MA, while the PD of the mDCP significantly decreased in the whole image and its subregions, except for the fovea. There was little difference between the two groups for the PD in the RPC, except for the PD inside the disk. Additionally, our results demonstrated a considerably reduced thickness of the pRNFL on average as well as that of several peripapillary regions (hemi-superior, hemi-inferior superior, nasal, temporal, inferior-temporal, and inferior-nasal quadrants). The detailed forest plots (MA vs. HC) are shown in Supplementary File S5.

**Table 2:** Differences in OCT/OCTA measurements between MA and healthy controls.

Outcome variables			Number of studies	Number of eyes		WMD + 95% CI	Value of *p*	*I*^2^ test (%)	Egger’s test
				MA	HC				
FAZ	FAZ area		5	188	190	0.053 (0.033,0.073)	**0.000**	21.80%	0.178
	FAZ perimeter		2	62	62	0.332 (0.117,0.546)	**0.002**	0.00%	-
MPD	SCP	Whole image	3	118	120	−0.345 (−0.952,0.262)	0.265	48.90%	0.205
		Hemi-superior	3	118	120	−0.544 (−1.182,0.094)	0.095	20.30%	0.024
		Hemi-inferior	3	118	120	−0.482 (−1.138,0.175)	0.150	0.00%	0.203
		Fovea	3	118	120	−2.714 (−7.131,1.704)	0.229	78.80%	0.027
		Parafovea	3	118	120	−1.420 (−3.291,0.452)	0.137	64.10%	0.253
		Perifovea	2	58	64	−1.483 (−2.787,-0.179)	**0.026**	0.00%	-
	DCP	Whole image	3	118	120	−1.460 (−2.324,-0.595)	**0.001**	0.00%	0.573
		Hemi-superior	3	118	120	−1.257 (−1.961,-0.554)	**0.000**	40.00%	0.123
		Hemi-inferior	3	118	120	−1.081 (−1.818,-0.344)	**0.004**	0.00%	0.547
		Fovea	3	118	120	−4.045 (−8.144,0.053)	0.053	73.10%	0.145
		Parafovea	3	118	120	−1.193 (−1.875,-0.512)	**0.001**	0.00%	0.080
		Perifovea	2	58	64	−2.041 (−4.001,-0.082)	**0.041**	0.00%	-
RPD	Whole image		3	118	120	−2.031 (−4.927,0.866)	0.169	92.60%	0.288
	Inside disk		3	118	120	−2.602 (−4.135,-1.069)	**0.001**	0.00%	0.178
	Peripapillary	Average	3	118	120	−2.295 (−5.220,0.630)	0.124	88.00%	0.376
		Superior	3	96	102	−3.194 (−6.766,0.379)	0.080	89.90%	0.353
		Inferior	3	96	102	−1.748 (−3.550,0.055)	0.057	59.30%	0.006
		Temporal	4	156	158	−2.134 (−4.276,0.009)	0.051	85.50%	0.131
		Nasal	4	156	158	−0.924 (−2.408,0.560)	0.222	57.30%	0.421
pRNFL	Average		6	198	262	−6.087 (−9.856,-2.318)	**0.002**	68.80%	0.150
	Hemi-superior		1	28	34	−8.900 (−13.647,-4.153)	**0.000**	-	-
	Hemi-inferior		1	28	34	−6.000 (−9.796,-2.204)	**0.002**	-	-
	Superior		3	110	124	−12.053 (−21.441,-2.666)	**0.012**	86.20%	0.977
	Inferior		3	110	124	−7.979 (−14.983,-0.975)	0.026	86.20%	0.367
	Temporal		7	260	314	−4.388 (−6.097,-2.678)	**0.000**	0.00%	0.322
	Nasal		7	260	314	−8.089 (−12.564,-3.615)	**0.000**	74.80%	0.435
	Superior-temporal		3	120	160	−0.640 (−4.921,3.641)	0.770	0.00%	0.389
	Inferior-temporal		3	120	160	−8.803 (−13.405,-4.202)	**0.000**	7.20%	0.477
	Superior-nasal		3	120	160	−4.483 (−9.441,0.476)	0.076	0.00%	0.790
	Inferior-nasal		3	120	160	−7.428 (−12.842,-2.015)	**0.007**	0.00%	0.610

MA, migraine with aura; HC, healthy control; WMD, weighted mean difference; CI, confidence interval; FAZ, foveal avascular zone; MPD, macular perfusion density; RPD, radial peripapillary capillary perfusion density; pRNFL, peripapillary retinal nerve fiber layer; SCP, superficial capillary plexus; DCP, deep capillary plexus. The bold values means the difference between the two groups is statistically significant.

#### 3.3.2. Meta-analysis for MO versus healthy controls

We also performed a meta-analysis on the FAZ and pRNFL thickness and determined the PD of mSCP, mDCP, and RPC of MO eyes and healthy control eyes (Table 3). For FAZ parameters, the area and perimeter were significantly enlarged in patients with MO than those of the eyes of healthy controls. For the OCTA metrics, no significant difference was found in the PD of the macular and RPC regions, except for the PD of mDCP in the parafovea. Moreover, our results showed significantly reduced pRNFL thickness on average, as well as that of several peripapillary regions (hemi-superior, hemi-inferior superior, nasal, temporal, and superior-nasal quadrants). The detailed forest plots (MO vs. HC) are shown in Supplementary File S6.

**Table 3:** Differences in OCT/OCTA measurements between MO and healthy controls.

Outcome variables			Number of studies	Number of eyes		WMD + 95% CI	Value of *p*	*I*^2^ test (%)	Egger’s test
				MO	HC				
FAZ	FAZ area		6	278	279	0.058 (0.013,0.103)	**0.012**	73.90%	0.149
	FAZ perimeter		2	61	62	0.288 (0.068,0.508)	**0.010**	0.00%	-
MPD	SCP	Whole image	5	236	228	−0.424 (−1.434,0.586)	0.411	59.80%	0.134
		Hemi-superior	4	190	182	0.256 (−0.348,0.860)	0.406	38.30%	0.036
		Hemi-inferior	4	190	182	0.307 (−0.264,0.878)	0.292	28.40%	0.050
		Fovea	5	236	228	−0.272 (−1.530,0.986)	0.672	10.50%	0.160
		Parafovea	5	236	228	−0.648 (−1.823,0.526)	0.279	61.20%	0.335
		Perifovea	4	210	204	−0.396 (−1.576,0.783)	0.510	65.60%	0.313
	DCP	Whole image	5	236	228	−0.691 (−1.653,0.270)	0.159	0.00%	0.214
		Hemi-superior	4	190	182	−0.769 (−1.816,0.277)	0.150	0.00%	0.416
		Hemi-inferior	4	190	182	−0.356 (−1.345,0.632)	0.480	0.00%	0.163
		Fovea	5	236	228	−0.605 (−1.937,0.726)	0.373	32.00%	0.320
		Parafovea	5	236	228	−0.720 (−1.425,-0.015)	**0.045**	0.00%	0.062
		Perifovea	4	210	204	−0.767 (−1.871,0.337)	0.173	12.90%	0.166
RPD	Whole image		4	210	204	−1.128 (−2.595,0.340)	0.132	86.40%	0.248
	Inside disk		4	210	204	−0.333 (−1.124,0.457)	0.408	0.00%	0.203
	Peripapillary	Average	4	210	204	−0.638 (−1.866,0.590)	0.309	64.20%	0.098
		Superior	3	164	158	−1.467 (−4.077,1.142)	0.270	78.40%	0.190
		Inferior	3	164	158	−0.227 (−0.928,0.474)	0.526	46.90%	0.085
		Temporal	2	56	64	−3.237 (−6.485,0.010)	0.051	78.60%	-
		Nasal	2	56	64	−1.673 (−3.801,0.455)	0.123	0.00%	-
pRNFL	Average		9	487	435	−4.219 (−6.740,-1.698)	**0.001**	61.90%	0.354
	Hemi-superior		2	63	77	−5.413 (−9.362,-1.465)	**0.007**	0.00%	-
	Hemi-inferior		2	63	77	−4.463 (−8.400,-0.526)	**0.026**	0.00%	-
	Superior		6	293	292	−6.425 (−14.491,1.641)	0.118	88.10%	0.901
	Inferior		6	293	292	−4.824 (−10.122,0.474)	0.074	76.40%	0.771
	Temporal		10	555	482	−3.090 (−4.499,-1.681)	**0.000**	27.20%	0.820
	Nasal		10	555	482	−3.325 (−5.200,-1.450)	**0.001**	43.10%	0.628
	Superior-temporal		3	232	160	−0.369(−4.523,3.785)	0.862	29.60%	0.092
	Inferior-temporal		3	232	160	−1.877 (−10.866,7.112)	0.682	78.90%	0.043
	Superior-nasal		3	232	160	−6.430 (−11.007,-1.854)	**0.006**	0.00%	0.635
	Inferior-nasal		3	232	160	−3.358 (−8.176,1.460)	0.172	34.40%	0.903

MO, migraine without aura; HC, healthy control; WMD, weighted mean difference; CI, confidence interval; FAZ, foveal avascular zone; MPD, macular perfusion density; RPD, radial peripapillary capillary perfusion density; pRNFL, peripapillary retinal nerve fiber layer; SCP, superficial capillary plexus; DCP, deep capillary plexus. The bold values means the difference between the two groups is statistically significant.

#### 3.3.3. Meta-analysis for MA versus MO

The outcomes of the quantitative synthesis on the eyes with MA and MO included the FAZ, pRNFL thickness, and PD of the mSCP, mDCP, and RPC (Table 4). For all measures, there was no discernible difference between the two groups (*p* > 0.05).

**Table 4:** Differences in OCT/OCTA measurements between MA and MO.

Outcome variables			Number of studies	Number of eyes		WMD + 95%CI	Value of *p*	*I*^2^ test (%)	Egger’s test
				MA	MO				
FAZ	FAZ area		3	90	87	0.167 (−0.129,0.463)	0.268	0.00%	0.429
	FAZ perimeter		2	62	61	0.010 (−0.145,0.166)	0.898	15.20%	–
MPD	SCP	Whole image	2	58	56	−0.657 (−1.940,0.625)	0.315	0.00%	–
		Hemi-superior	2	58	56	−0.744 (−2.049,0.561)	0.264	0.00%	–
		Hemi-inferior	2	58	56	−0.580 (−1.877,0.718)	0.381	0.00%	–
		Fovea	2	58	56	−1.460 (−4.318,1.399)	0.317	0.00%	–
		Parafovea	2	58	56	−1.682 (−3.473,0.108)	0.066	0.00%	–
		Perifovea	2	58	56	−0.330 (−1.769,1.110)	0.654	0.00%	–
	DCP	Whole image	2	58	56	−0.702 (−3.127,1.723)	0.571	0.00%	–
		Hemi-superior	2	58	56	−1.461 (−3.861,0.939)	0.233	0.00%	–
		Hemi-inferior	2	58	56	−0.635 (−2.920,1.650)	0.586	0.00%	–
		Fovea	2	58	56	−2.536 (−5.520,0.448)	0.096	0.00%	–
		Parafovea	2	58	56	−0.804 (−2.740,1.132)	0.416	0.00%	–
		Perifovea	2	58	56	−0.511 (−2.872,1.850)	0.671	0.00%	–
RPD	Whole image		2	58	56	−0.656 (−1.730,0.419)	0.232	0.00%	–
	Inside disk		2	58	56	−1.450 (−3.475,0.575)	0.16	0.00%	–
	Peripapillary	Average	2	58	56	−1.323 (−2.973,0.327)	0.116	0.00%	–
		Superior	2	58	56	−1.896 (−3.858,0.065)	0.058	0.00%	–
		Inferior	2	58	56	−1.160 (−2.751,0.430)	0.153	0.00%	–
		Temporal	2	58	56	−1.037 (−2.435,0.361)	0.146	0.00%	–
		Nasal	2	58	56	−1.151 (−3.283,0.981)	0.29	0.00%	–
pRNFL	Average		5	160	268	−1.847 (−3.936,0.242)	0.083	0.00%	0.398
	Superior		3	110	111	−2.584 (−6.651,1.482)	0.213	0.00%	0.887
	Inferior		3	110	111	−0.960 (−4.136,2.215)	0.553	0.00%	0.873
	Temporal		7	260	373	−1.314 (−3.078,0.450)	0.144	0.00%	0.094
	Nasal		7	260	373	−3.244 (−8.186,1.698)	0.198	81.20%	0.729
	Superior-temporal		3	120	232	0.048 (−4.725,4.821)	0.984	0.00%	0.028
	Inferior-temporal		3	120	232	−4.089 (−8.540,0.361)	0.072	10.30%	0.380
	Superior-nasal		3	120	232	1.584 (−2.874,6.042)	0.486	0.00%	0.891
	Inferior-nasal		3	120	232	−4.176 (−9.702.1.350)	0.139	0.00%	0.823

MA, migraine with aura; MO, migraine without aura; WMD, weighted mean difference; CI, confidence interval; FAZ, foveal avascular zone; MPD, macular perfusion density; RPD, radial peripapillary capillary perfusion density; pRNFL, peripapillary retinal nerve fiber layer; SCP, superficial capillary plexus; DCP, deep capillary plexus.

### 3.4. Sensitivity analysis and subgroup analysis

We performed sensitivity analysis by removing one study in each of the direct comparisons. When we omitted Karahan et al. (24), the five comparisons (fovea PD in mSCP, parafovea PD in mSCP, fovea PD in mDCP, peripapillary average PD in RPC, and peripapillary-nasal PD in the RPC) became statistically significant (*p* < 0.05). For the peripapillary-superior and nasal PD in the RPC, the removal of Hamurcu et al. (22) contributed the most to the heterogeneity. However, their absence had no impact on the outcomes. The detailed sensitivity analysis is shown in Supplementary File S7.

After a thorough review of the characteristics, we next performed a subgroup analysis according to the age of patients and OCT/OCTA devices. The results showed that the age and the OCT/OCTA device were the sources of heterogeneity in several comparisons. The detailed subgroup analysis is provided in Supplementary File S8.

### 3.5. Publication bias

In the majority of the comparisons, Egger’s tests revealed that there was no publication bias (*p* ≥ 0.05), except for the analysis of the PD of the mSCP in the hemi-superior and fovea regions (MA vs. HC), the PD of the RPC in the peripapillary-inferior quadrant (MA vs. HC), and the pRNFL thickness in the inferior-temporal quadrant (MO vs. HC). Egger’s test results are shown in Tables 2–4.

## 4. Discussion

The present meta-analysis utilized OCT and OCTA parameters, such as pRNFL thickness, FAZ area, MPD, and PD of RPC, to analyze the retina and microvascular alterations between MA and healthy controls, MO and healthy controls, and MA and MO eyes. The main results are as follows: (1) Compared to healthy controls, the thickness of the pRNFL of patients with MA significantly decreased in most areas except in the inferior, superior-temporal, and superior-nasal quadrants, while the area and circumference of the FAZ significantly increased. The PD of mDCP in the patients with MA significantly decreased in the whole image and its subregions except for the fovea. The PD of the RPC also significantly decreased inside the disk. (2) The average thickness of pRNFL of the patients with MO, as well as that of several peripapillary regions, decreased, while the FAZ parameters significantly increased. The PD of the mSCP, mDCP, and RPC was not significantly different, except for the mDCP in the parafovea. (3) No statistical significance was observed in the retina and microvascular features of the patients with MA and MO. These results suggest that there is a certain degree of optic nerve and retinal microvascular damage in patients with migraine, especially those with MA.

The thinning of RNFL thickness in patients with migraine was first reported by Martinez et al. in 2008 (34) The thinned RNFL was mostly located in specific quadrants around the optic nerve papillae. The present meta-analysis found a significant decrease in the average pRNFL thickness in patients with migraine, consistent with most of the previous studies. Some possible causes for this decrease include abnormal vascular changes such as abnormal vascular regulation and focal cerebral ischemia. To explain the above, Kara et al., in as early as 2003, applied color Doppler ultrasound and observed that the blood flow in the central retinal and posterior ciliary arteries was lower in patients with migraine than that in normal healthy subjects (35). Migraine aura or attack can change the retinal and nerve blood supply, which can lead to ischemic and hypoxic injury, which may in turn damage the retinal nerve. pRNFL is composed of nerve fibers consisting of retinal ganglion cell axons, which are mostly unmyelinated and require more energy supply and therefore are more vulnerable to retinal ischemic injury (36). The present meta-analysis found significant quadrant-specific pRNFL thinning in patients with migraine, concentrated in the superior hemisphere, inferior hemisphere, and temporal and nasal quadrants. This selective pRNFL involvement may be related to differences in the retinal ganglion cell axon sensitivity to the local ischemia and focal perimetric changes. The abnormal blood supply to the fundus due to migraine occurs mostly in these specific regions (37).

The FAZ is a region jointly delineated by both superficial and deep capillaries. This region is round or oval in healthy individuals, with an area of approximately 0.231–0.280 mm^2^. Any changes in its size and shape can indirectly lead to macular microvascular changes (38, 39). Previous studies have shown that the FAZ enlarges in various systemic diseases (40). However, changes in the FAZ have not been clearly established in patients with migraine. The meta-results showed a significant increase in the area and circumference of the FAZ in patients with migraine, including those with MA and MO, which may be closely related to retinal ischemia caused by vasospastic alterations in these patients. Recurrent attacks of migraine in these patients cause transient spasms of intracranial and intraocular vessels, resulting in acute and chronic microvascular and perfusion changes in retinal vessels, with the consequent enlargement of the FAZ area and circumference. However, clinical studies have also yielded inconsistent results. Kurtul et al. (17) found no significant changes in the FAZ of patients with migraine. Karahan et al. (24) found changes only in the deep FAZ area in patients with MA. These results suggest that there are individual differences in the FAZ size, which are closely related to various factors such as the macular central recess structure, eye axis length, age, race, and gender (41).

To avoid conceptual confusion, we uniformly applied PD to express the ratio between the blood flow area to the total scan area. A major advantage of OCTA over conventional fundus fluoroscopy is that the former allows quantitative measurement of the macular and optic papillary perfusion density. The present meta-analysis showed that compared with healthy controls, the PD of the mDCP in patients with MA significantly decreased in the whole image and its subregions except for the fovea, which may be closely related to the retinal ischemia caused by MA. The development of MA symptoms is currently considered to be a clinical manifestation of cortical spreading depression (CSD), a depolarizing wave that propagates along the cerebral cortex and has an inhibitory effect on the cortical function, which increases the cortical cerebral blood flow and disturbances in neurovascular coupling. Studies have shown that CSD during MA episodes can lead to a change from an initial transient increase in the blood flow to hypoperfusion changes in the cerebral cortex. However, its electrophysiological correlation with ocular vascular nerves has not been confirmed (1, 42). Based on its retinal localization propagation over the visual cortex, the characteristics of visual defects and imaging studies indirectly suggest a correlation with the ocular vasculature (43). Therefore, the above discussion could indicate the presence of intracranial and ocular retinal microvascular injuries and ischemic changes in patients with MA. In the PD of the RPC, our current meta-analysis found almost no significant difference between patients with migraine and healthy controls. A further sensitivity analysis showed significant differences between MA and HC in the average PD of the RPC and in some specific quadrants (superior, inferior, and nasal) after excluding Hamurcu et al. (22) and Karahan et al. (24). Hence, it can be suggested that patients with MA show some degree of reduced PD of the RPC, while the overall difference is not statistically significant. Hence, the optic nerve papilla could be more resistant to ischemia, hypoxia, and inflammation than the central macular sulcus (17). In addition, the small sample size included in the study could explain why PD in the RPC appears unaffected.

The current meta-analysis further compared the retinal and microvascular characteristics of the two subgroups of MA and MO and found that patients with MA had an enlarged FAZ area and circumference and a reduced macular and optic disk PD. However, the differences were not statistically significant, which is somewhat different from the previous meta-analysis results (44). The posterior regions of the cerebral hemispheres were usually more significantly malperfused during migraine attacks in patients with MA compared to patients with MO (45). The lack of statistical difference between the two in the current meta-analysis could be owing to various factors such as the sample size of the included studies, migraine variables, and ethnic differences. Hence, large prospective cohort studies are needed for validating the results of this meta-analysis.

This is a comprehensive assessment of the retina and microvascular alterations in patients with MA and MO using systematic review and meta-analysis, providing an evidence-based basis for the characteristic fundus manifestations of migraine. Compared with previous literature published by Ke W et al. (46), this meta-analysis further compares the thickness of the pRNFL and the optic disk PD to evaluate the retina and optic microvascular alterations. However, the study has some limitations: (1) Sensitivity analysis: exclusion of literature such as Karahan et al. (24) and Hamurcu et al. (22) can lead to alterations in some macular and optic disk PD results; therefore, caution should be taken with the above meta-analysis results. (2) Careful sensitivity analysis and subgroup analysis showed that some comparisons had large heterogeneity possibly because of the sample size, ethnic differences, and migraine variable correlations. (3) Owing to the lack of information in the literature, factors that may affect OCTA measurements were not analyzed. (4) The majority of the studies included in the current meta-analysis were from Turkey, and there is a certain bias, so the results should be analyzed with caution. (5) The sample size of some comparisons was relatively small, which could cause a bias. (6) Few articles reported systemic diseases that may affect retinal microcirculation. Therefore, we could not analyze these potential confounding factors.

## 5. Conclusion

The eyes of patients with MA and MO demonstrated significant pRNFL thickness impairments in some regions. Patients with MA showed retinal microvascular impairments, including FAZ enlargement and decreased PD in the mDCP. An enlarged FAZ in patients with MO was also shown. The OCT and OCTA could detect membrane morphology and circulation status in migraine and might provide the basis for the diagnosis and follow-up of patients with migraine.

## Data availability statement

The original contributions presented in the study are included in the article/Supplementary material, further inquiries can be directed to the corresponding author.

## Author contributions

ZL: conceptualization, methodology, formal analysis, and writing the original draft. XH and YD: methodology and supervision. WZ, JYW, and YL: visualization and review editing. JWW: language editing and supervision. CJ: conceptualization, funding, and project administration. All authors contributed to the article and approved the submitted version.

## Funding

This study was supported by grants from the National Natural Science Foundation of China (general program No. 81874494), the Science and Technology Innovation Project of China Academy of Chinese Medical Sciences (No. CI2021A02604), the Natural Science Foundation of Beijing Municipality (No. 7232325), Beijing Traditional Chinese Medicine Technology Development Fund (No. BJZYYB-2023-57).

## Conflict of interest

The authors declare that the research was conducted in the absence of any commercial or financial relationships that could be construed as a potential conflict of interest.

## Publisher’s note

All claims expressed in this article are solely those of the authors and do not necessarily represent those of their affiliated organizations, or those of the publisher, the editors and the reviewers. Any product that may be evaluated in this article, or claim that may be made by its manufacturer, is not guaranteed or endorsed by the publisher.
